# Lineage plasticity and immune invisibility in primary SCLC and LUAD-to-SCLC transformation: antigen presentation loss and therapeutic redirection

**DOI:** 10.3389/fimmu.2026.1900927

**Published:** 2026-09-04

**Authors:** Hongying Xu, Benhua Li, Jun Yang, Xin Tang

**Affiliations:** 1 Department of Oncology, The First Affiliated Hospital of Chongqing Medical and Pharmaceutical College, Chongqing, China; 2 Department of Clinical Laboratory, The Second People’s Hospital of Liangshan Yi Autonomous Prefecture, Xichang, China; 3 Department of Respiratory and Critical Care Medicine, People’s Hospital of Chongqing Liangjiang New Area, Chongqing, China

**Keywords:** antigen presentation, immune checkpoint blockade, immune invisibility, lineage plasticity, MHC-I, neuroendocrine transformation, small-cell lung cancer, therapeutic redirection

## Abstract

Immune checkpoint inhibitors have improved outcomes in small-cell lung cancer (SCLC), yet durable benefit remains limited, and the immune consequences of SCLC transformation from EGFR-mutant lung adenocarcinoma (LUAD) remain incompletely understood. Conventional explanations for resistance emphasize PD-L1 expression, tumor mutational burden, T-cell dysfunction, myeloid suppression, and stromal exclusion, but these factors do not fully explain how lineage-state changes alter tumor recognition. Here, we examine lineage plasticity as a potential tumor-cell-intrinsic contributor to immune resistance. We use the term “immune invisibility” narrowly to denote failure of tumor-cell recognition caused primarily by impaired MHC-I antigen processing and presentation. This state is distinguished from immune exclusion and T-cell dysfunction, although the three barriers may coexist. Evidence linking neuroendocrine state to reduced antigen presentation is strongest in primary SCLC and experimental SCLC models. In EGFR-mutant lung adenocarcinoma undergoing SCLC transformation, analogous changes remain biologically plausible but have not been established in paired longitudinal specimens. Therapeutically, checkpoint blockade alone may be insufficient when tumor recognition or T-cell entry is impaired. Clinically established approaches include chemoimmunotherapy in defined SCLC settings and DLL3-directed T-cell engagement in previously treated disease, whereas epigenetic combinations, STING/IFN activation, most antibody-drug conjugates, and cellular therapies remain investigational. Integrating lineage state, antigen-presentation capacity, immune localization, and retained surface targets may provide a framework for distinguishing immune-restoration from immune-redirection strategies.

## 1 Introduction

Immune checkpoint inhibition changed the treatment paradigm of lung cancer in every disease stage and in almost all histologic subtypes. In advanced non-small cell lung cancer (NSCLC), PD-1/PD-L1 blockades proved to improve survival as monotherapy in selected PD-L1-high tumors and in combination with chemotherapy. And dual checkpoint blockade as well as consolidation durvalumab were able to extend immune-based therapy into metastatic and inoperable locally advanced stage III disease (1–5). In small-cell lung cancer (SCLC), atezolizumab or durvalumab combined with platinum-etoposide entered first-line treatment for patients with extensive-stage disease (6–8). But this progress is far away from benefiting everyone and especially there seems still no convincing evidence showing positive effects of immunotherapy in patients carrying targetable oncogenic driver mutations of NSCLC, transformed SCLCs and neuroendocrine tumors.

At present, explanations of why some patients fail to benefit are most commonly focused on PD-L1 expression status, tumor mutational burden, T cell exhaustion, myeloid suppression, stromal exclusion, and metabolic constraint within the tumor microenvironment (TME). EGFR-mutant and ALK-rearranged NSCLCs have shown very low rates of response to PD-1/PD-L1 blockade, which is reflected in their low levels of PD-L1 expression and reduced CD8^+^ T-cell infiltration (9). But more broadly speaking, responses can also be predicted by tumor mutational burden, PD-L1 expression and T-cell-inflamed gene expression profiles (10–12). However, none of these metrics appear able to predict response across all lung cancer subtypes. Thus it seems that one cannot determine immune sensitivity simply through measurement of antigen load, but must also consider if cancer cells will actually present antigen, recruit T cells and maintain a tissue environment capable of sustaining immune activation.

There is increasing evidence that tumor-cell identity can influence immune recognition. Lineage plasticity, the capacity of cancer cells to change their commitment status, thus acquiring another lineage phenotype, is now recognized as one of the major mechanisms responsible for adaptive therapy responses and the formation of resistant clones (13, 14). There are many examples of lineage plasticity in lung cancer, such as histologic conversion events, which are mostly seen when lung adenocarcinomas convert to SCLC-like neuroendocrine carcinoma, squamous-like states, mixed histology or poorly differentiated phenotypes following treatment (15–17). Cancer cells with coexisting EGFR, TP53 and RB1 alterations represent a patient group having higher chances to undergo conversion into small-cell lung cancers, but other factors may also play roles because there is growing evidence showing that these events happen only when the cells have developed tolerance to certain onco-states incompatible with their own lineage properties (18, 19).

The immunologic consequence of lineage plasticity becomes increasingly relevant here too. Again, there is a fundamental contradiction in the case of small cell lung cancer (SCLC); while there is frequent association with smoking along with high mutation burden, most patients do not get long-lasting advantage from immune checkpoint inhibitors (ICIs). But comprehensive genomic investigations have indeed found SCLC highly altered genetically, almost every tumor having TP53 and RB1 both compromised, yet this degree of mutation load does not automatically make the patient more responsive to ICIs (20). Nor is it correct to think of SCLC as being entirely immune-cold either; there exist multiple neuroendocrine, non-neuroendocrine and inflamed subsets instead. And the SCLC-I subset, YAP1-associated tumors and also several additional pieces of recent evidence obtained directly through clinical trials show how the lineage state of a tumor, its relation to T-cell infiltration or to infiltration by different kinds of macrophages can make it more likely or less likely to respond if combined treatment with PD-L1 blockade together with chemo is used (21–23).

Antigen presentation provides a key bridge between lineage plasticity and immune response. A CheckMate 032-based analysis showed that clinical benefit from immune checkpoint blockade in SCLC was associated with tumor antigen presentation capacity rather than conventional transcriptional subtype alone (24). Experimental work further showed that SCLC can contain non-neuroendocrine MHC-I-high cell states associated with greater immune recognition and durable benefit from immune checkpoint blockade (25). These findings suggest that lineage plasticity can either suppress or expose tumor immunogenicity depending on the direction and stability of cell-state transition. Neuroendocrine-high or SCLC-like states may reduce MHC-I expression and weaken CD8^+^ T-cell recognition, whereas non-neuroendocrine or inflamed-like states may restore partial immune visibility.

**Figure 1:**
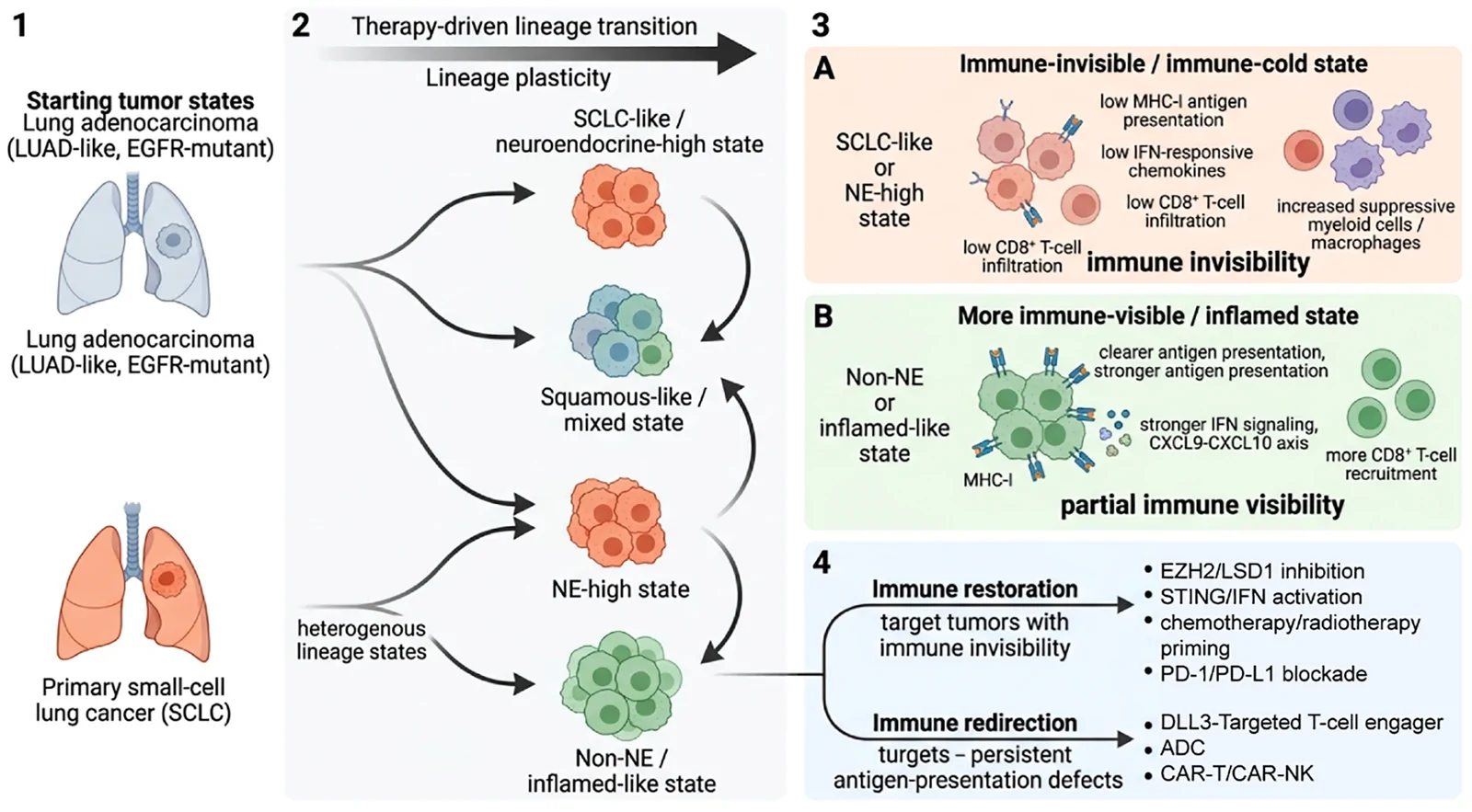
Proposed relationships between lineage plasticity and immune resistance in lung cancer. Lung cancer cells can transition from LUAD-like states toward SCLC-like or neuroendocrine-high states under therapeutic pressure. Primary SCLC also contains heterogeneous neuroendocrine-high, non-neuroendocrine, and inflamed-like states. Lineage state may influence several distinct levels of immune resistance. Impaired MHC-I antigen processing and presentation can reduce tumor-cell recognition and produce immune invisibility, whereas weak chemokine networks or stromal barriers can produce immune exclusion, and suppressive myeloid or stromal signals can produce local T-cell dysfunction. Evidence for lineage-associated antigen-presentation differences is strongest in primary SCLC and experimental SCLC models. The immune consequences of squamous-like and mixed-lineage transitions remain incompletely defined. Depending on the dominant barrier, treatment may aim to restore endogenous immune recognition or redirect immune effectors through retained surface targets. Created in BioRender. https://BioRender.com/zxjidl0.

In this review, we focus on neuroendocrine lineage plasticity and immune resistance in two related but biologically distinct settings: primary SCLC and SCLC transformation from EGFR-mutant LUAD. We define “immune invisibility” narrowly as failure of tumor-cell recognition caused primarily by impaired MHC-I antigen processing and presentation. This state is distinct from immune exclusion, in which tumor-reactive lymphocytes fail to enter or approach malignant-cell nests, and from T-cell dysfunction, in which infiltrating T cells are rendered ineffective by chronic antigen exposure, inhibitory signaling, metabolic stress, or suppressive stromal and myeloid cells. These barriers may coexist and interact, but they represent different biological levels of immune resistance. Direct evidence linking lineage state to impaired antigen presentation is strongest in primary SCLC and experimental SCLC models. Applying this relationship to EGFR-mutant lung adenocarcinoma after SCLC transformation remains a mechanistically supported hypothesis that requires validation in paired pre- and post-transformation specimens. We therefore distinguish failure of tumor recognition from defective immune-cell entry and local immune dysfunction, and then consider whether therapy should restore endogenous recognition or redirect immune effectors through retained surface targets. This framework is summarized in **Figure 1**.

### 1.1 Literature search strategy

We searched PubMed, Web of Science, and Scopus from database inception to April 2026 using combinations of the terms “lung cancer,” “lineage plasticity,” “neuroendocrine transformation,” “small-cell transformation,” “SCLC,” “MHC-I,” “HLA-I,” “antigen presentation,” “immune evasion,” “immune exclusion,” “DLL3,” “T-cell engager,” and “NK cell.” English-language clinical studies, trial-associated translational analyses, preclinical studies, and major reviews relevant to lung cancer lineage state, SCLC transformation, immune recognition, or immune-directed therapy were considered. Priority was given to paired clinical specimens, clinical-trial-associated datasets, and studies directly examining SCLC. Evidence from other tumor types was included only as mechanistic context and was distinguished from direct evidence in transformed SCLC.

## 2 Clinical settings: a place where lineage plasticity comes together with immune resistance

Lineage plasticity is most apparent when a tumor changes its histologic or transcriptional identity during treatment (13, 14). In lung cancer, two settings are central to the present review: EGFR-mutant LUAD undergoing SCLC transformation (15–19) and lineage and immune heterogeneity within primary SCLC (20–26). Direct evidence linking lineage state to antigen-presentation capacity and immune engagement is strongest in primary SCLC (21–25), whereas comparable longitudinal immune profiling before and after SCLC transformation remains limited. Squamous-like, mixed and dedifferentiated transitions illustrate the broader spectrum of lung cancer plasticity, but their immune consequences remain less well defined (27, 28).

### 2.1 EGFR-mutant LUAD after SCLC transformation

A well-established example of lineage plasticity in lung cancer is the transformation of EGFR-mutant LUAD into SCLC or SCLC-like neuroendocrine carcinoma during acquired resistance to EGFR-targeted therapy. Early paired-biopsy studies showed that transformed tumors retained the original activating EGFR mutation despite marked histopathological conversion, supporting clonal continuity between the pre-existing adenocarcinoma and the transformed SCLC rather than the development of an independent second primary tumor (15).

In general, transformed tumors adopt neuroendocrine morphology and express markers such as synaptophysin, chromogranin A or CD56 whereas EGFR expression and dependence become reduced (16). RB loss is particularly enriched in EGFR-mutant cancers that transform to SCLC, suggesting that disruption of lineage-stability checkpoints may create a permissive background for neuroendocrine transition (16, 18). When analyzing data from several centers together, EGFR-mutant adenocarcinomas which became transformed to SCLC or other neuroendocrine carcinomas retained the original EGFR mutation but behaved very aggressively. Thus transformed SCLC appears to represent a distinct acquired-resistance state (17).

But also note that there is variation in the extent of risk transformation within EGFR-mutated LUAD, co-alterations in EGFR, TP53 and RB1 identify a subset of patients carrying a higher risk of occurrence of histologic transformation toward small-cell lung cancer type, together with worse outcomes overall (18); which therefore indicates that TP53/RB1 inactivation is not only something occurring later on during the process of change, but actually constitutes a pre-existent backdrop upon which treatment pressure triggers cell state conversion; while experimental models additionally show that for transformation to happen cells need some kind of state-permissive status such as tolerance toward the activation of oncogenic programs incompatible with the original cell-lineage identity (19).

From an immune perspective, transformed SCLC may combine two potentially unfavorable backgrounds. EGFR-mutant LUAD generally shows limited sensitivity to PD-1/PD-L1 blockade (9), whereas neuroendocrine-high primary SCLC states are often associated with weaker antigen-presentation and inflammatory programs (21, 24, 25). It is therefore biologically plausible, but not yet directly established, that acquisition of an SCLC-like phenotype further reduces immune visibility. Direct paired analyses demonstrating progressive loss of MHC-I expression, IFN responsiveness or T-cell infiltration during LUAD-to-SCLC transformation remain scarce. Transformed SCLC should consequently be viewed as a distinct evolutionary state shaped by the founder LUAD clone, treatment-induced selection, TP53/RB1-associated lineage instability and acquisition of neuroendocrine features (15–19), while its immune phenotype requires direct longitudinal characterization.

### 2.2 Primary SCLC as a plastic and immune-heterogeneous disease

In turn, primary SCLC gives another scenario where lineage state meets up with immune resistance. In terms of their genome composition, nearly all SCLCs carry mutations leading to loss-of-function of TP53 and RB1 and recurrent changes affecting NOTCH signaling, MYC family genes and chromatin regulators (20). While SCLC has typically been considered one type of high-grade neuroendocrine carcinoma, recent genomic and transcriptomic analyses show that SCLC consists of distinct lineage states instead of maintaining just one single entity (20, 26).

Several partially overlapping classification schemes are used in SCLC. SCLC-A, SCLC-N, and SCLC-P are defined primarily by ASCL1-, NEUROD1-, and POU2F3-dominant tumor-cell transcriptional programs, respectively (21). By contrast, SCLC-I is an immune-enriched transcriptional state characterized by low expression of canonical lineage transcription factors rather than a directly equivalent lineage category. Immunohistochemical studies confirm heterogeneity in ASCL1, NEUROD1, POU2F3, and YAP1 expression in clinical specimens (29), while POU2F3 identifies a tuft-cell-like transcriptional program in a subset of tumors (30). Neuroendocrine differentiation, MHC-I activity, and immune-cell infiltration should therefore be assessed as related but separate biological variables rather than inferred directly from a subtype label.

Within the transcriptional framework proposed by Gay and colleagues, the SCLC-I group showed the greatest relative benefit from adding immunotherapy to chemotherapy, linking an inflamed state to therapeutic sensitivity (21). YAP1-associated tumors have also been reported to display T-cell-inflamed features, although YAP1 expression should not be treated as a universally accepted standalone subtype (22). More recent trial-associated analyses show that immune-inflamed tumors occur across neuroendocrine and non-neuroendocrine contexts and that the balance between T-effector and macrophage-associated signals may influence vulnerability to PD-L1 blockade plus chemotherapy (23).

There is additional support for SCLC plasticity coming from both developmental and experimental models. There is evidence, for example, that NOTCH signaling may give rise to non-neuroendocrine tumor-cell states within SCLC, thereby driving intratumoral heterogeneity (31). And there is now emerging evidence from recent lineage-tracing and modeling studies which show that SCLC as well as associated neuroendocrine-tuft tumors can originate from cell states with greater lineage potential than was previously thought possible. Again therefore there seems to be good reason to view the current array of SCLC subtypes simply as dynamic lineage trajectories rather than fixed categories (32).

**Figure 2:**
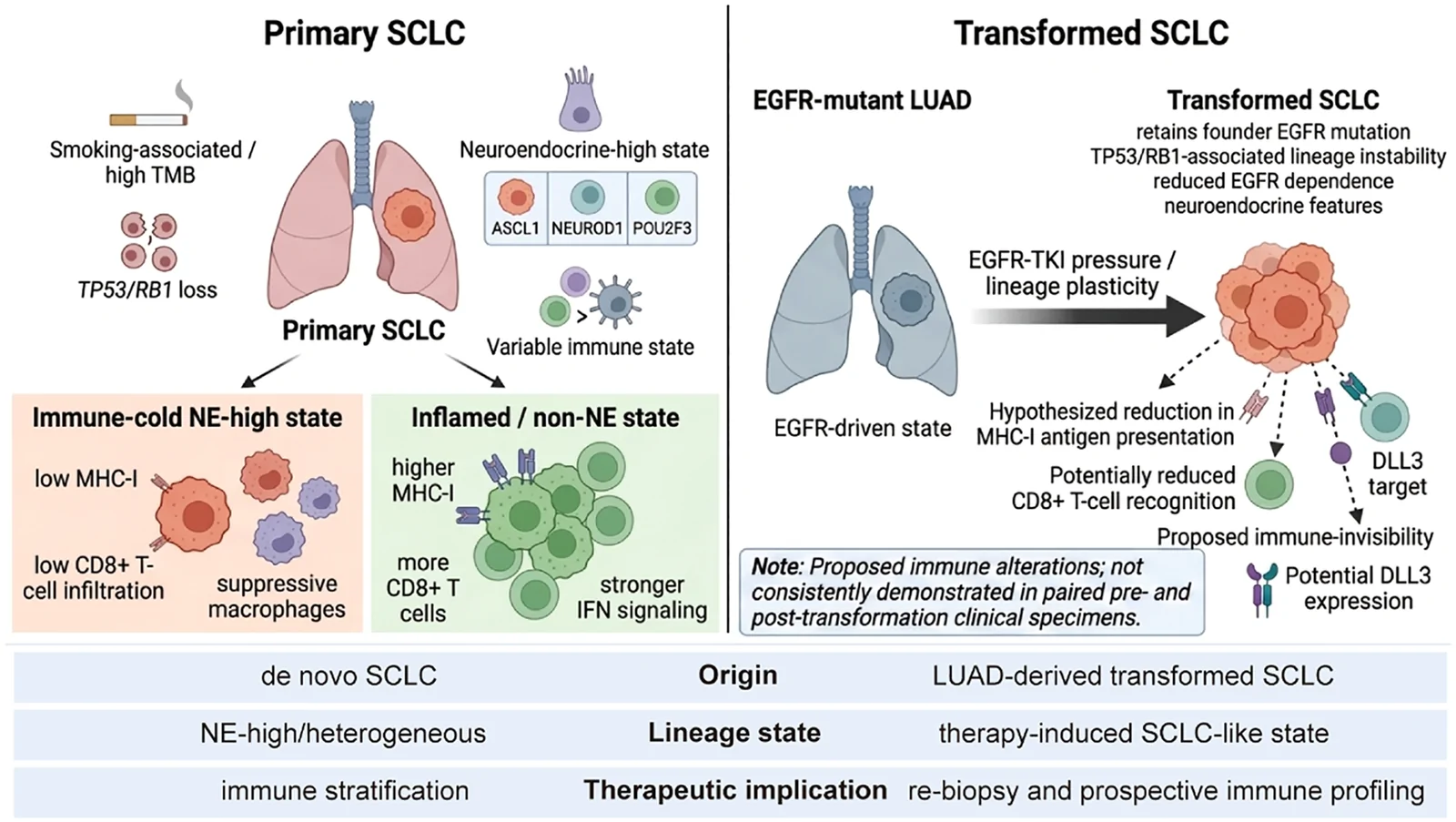
Distinct origins and proposed immune features of primary and transformed SCLC. Primary and transformed SCLC share neuroendocrine morphology and frequent TP53/RB1 pathway disruption but arise through different biological routes. Primary SCLC develops *de novo* as a high-grade neuroendocrine carcinoma and spans heterogeneous neuroendocrine-high, non-neuroendocrine, and immune-inflamed states. Transformed SCLC arises from a pre-existing lung adenocarcinoma, commonly during EGFR-targeted therapy, retains the founder driver alteration, and acquires SCLC-like morphology with reduced EGFR dependence. Direct longitudinal evidence showing that transformation is consistently accompanied by MHC-I loss, impaired IFN responsiveness, or T-cell exclusion remains limited. Primary and transformed SCLC should therefore not be treated as immunologically equivalent entities, supporting tissue re-biopsy and prospective immune profiling at progression. The immune alterations illustrated in the transformed-SCLC panel represent biologically supported hypotheses extrapolated mainly from primary SCLC and experimental SCLC models, rather than established and uniform consequences of transformation. Created in BioRender. https://BioRender.com/hmg16zg.

Collectively, primary SCLC is better understood as a continuum of lineage and immune states than as a uniformly immune-cold endpoint (21, 23–26). Neuroendocrine-high tumors often show weak antigen-presentation programs and limited T-cell recognition, whereas selected non-neuroendocrine or inflamed tumors retain greater immune engagement (21, 24, 25). Importantly, transcriptional subtype, neuroendocrine differentiation, antigen-presentation capacity, and immune-cell infiltration are not interchangeable classification systems. Their relationships should be measured directly and interpreted within the clinical and spatial context of each tumor. **Figure 2** summarizes the distinct biological origins and current evidence for the immune states of primary and transformed SCLC.

### 2.3 Other lineage transitions: current evidence and limitations

Although neuroendocrine transformation is the principal focus of this review, lineage switching in lung cancer is not restricted to SCLC-like states. Tissue-based analyses of acquired resistance to first-line osimertinib identified squamous transformation as an early off-target resistance mechanism associated with greater genomic complexity and poor clinical outcomes (27). Histologic transformation in EGFR-mutant LUAD may also include large-cell neuroendocrine carcinoma, sarcomatoid features and combined phenotypes, further illustrating that lung cancer can occupy intermediate or hybrid lineage states (28).

However, available studies of these non-SCLC transitions have focused mainly on histologic evolution and treatment resistance rather than immune phenotyping (27, 28). It therefore remains unclear whether squamous-like, mixed or dedifferentiated states reproducibly alter MHC-I expression, IFN-responsive signaling, immune-cell localization or lineage-associated surface-target expression. These states are included here to define the broader scope of lineage plasticity, but they are not treated as established examples of lineage-associated immune invisibility. Accordingly, the mechanistic and therapeutic framework developed below focuses principally on primary SCLC and SCLC-like neuroendocrine transformation.

## 3 Loss of antigen presentation: the core of lineage-related immune invisibility

Cytotoxic CD8^+^ T cells need to see MHC-I antigen-presenting tumor cells first. Therefore, for them to recognize tumor cells, tumor antigens must be processed and presented via the MHC-I antigen presentation pathway which involves proteasomal antigen generation, then transport by TAP1 and TAP2 followed by loading onto HLA class I molecules together with β2-microglobulin before becoming stably displayed on the surface of tumor cells as peptide-MHC-I complexes. Any weakening of this pathway will make tumor cells having kept their genetic alterations and potential neoantigens while being seen less by T cells functionally. It has been shown loss of MHC-I antigen presentation is one important form of cancer immune evasion and also blocks T-cell based immunotherapy (33–36).

The above finding may be significant because the discussion about lung cancer immunotherapy very often starts from tumor mutational burden (TMB). Higher nonsynonymous mutation burden and also neoantigen load in NSCLC are positively correlated with better response rates to PD-1 blockade, supporting the hypothesis that the mutation-derived antigens indeed help sensitize tumors to immunotherapy (10). But mutations do not automatically mean immune recognition-the tumor antigens need to be presented in the right way to meet T-cell receptors, which means intact antigen processing and MHC-I expression, both of which are prerequisites of successful immune recognition. Even if clonal neoantigens drive immune surveillance, their effect will inevitably be weakened once the tumor cells lose antigen presentation ability or if antigen presentation becomes spatially restricted (34, 37, 38).

In lung cancer, alteration of HLA-I itself is known to be recurrently involved in immune evasion too. First of all, haplotype loss of HLA class I antigens has been described as an immune escape mechanism in lung cancer, thus indicating that also partial reduction of HLA-I molecules could allow tumor cells to evade the recognition by cytotoxic T-cells (39). Furthermore, TRACERx analyses revealed that allele-specific HLA loss occurs during NSCLC evolution, being possibly caused by immune selection pressure (38). In addition, acquired B2M loss can interfere with proper HLA-I expression on cell surface leading to acquired resistance against PD-1 blockade in both lung cancer models and other checkpoint-treated cancers (40, 41). Finally, spatial analysis performed on NSCLC specimens suggested that there is variability inside tumors regarding both HLA class I and class II subunits’ expression which turned out to have clinical relevance as well: so this fact strengthens even more the necessity to study antigen presentation taking into account the tissue context instead of considering just one global characteristic for entire tumor sample (42). All these results allow us now to say that we have enough evidence about this matter that antigen presentation impairment through alteration or loss of HLA-I antigens might represent also in lung cancer an actual clinically-relevant immune escape mechanism instead of simply being a hypothetical possibility previously hypothesized from researchers.

SCLC provides a particularly informative example of the disconnect between neoantigen availability and effective tumor recognition. Because of its frequent association with smoking history, along with its relatively high mutation rate, most people expect this cancer has plenty of neoantigens. Yet even here, where neoantigen supply seems plentiful, durable responses to single-agent PD-L1 or PD-1 inhibitors happen mostly in just some patients-usually those who had already responded temporarily to prior platinum chemotherapy (20). Despite the high mutational burden of SCLC, mutation load alone does not reliably predict durable benefit from immune checkpoint blockade (26, 43). Studies conducted many years ago found very poor levels of HLA class I expression within SCLC cell lines (44). Taken together then, there’s reason to think SCLCs might usually suffer from some sort of bottleneck occurring somewhere between neoantigen formation itself on the one hand, and full recognition of said neoantigens via activated CD8+ cytotoxic lymphocytes on the other-and that often enough what limits things will end up being inadequate antigen presentation capacity rather than insufficient antigen availability.

In support of this conclusion is clinical data. In an evaluation of pretreatment tumor samples collected in CheckMate 032, which studied nivolumab given either alone or together with ipilimumab in SCLC patients, durable clinical benefit was found to be associated with tumor antigen presentation capacity (24). Significantly, conventional SCLC transcriptional subtype alone did not appear able to account fully for benefit from immune checkpoint blockade, indeed, it appeared that whether tumor cells could actually present antigens might matter more than what their lineage designation happened to be (24). Thus there does seem good reason for putting emphasis on antigen presentation when thinking about the immune invisibility associated with lineage.

Neuroendocrine differentiation and antigen-presentation activity are related but non-equivalent variables. Neuroendocrine-high states often express weaker antigen-presentation programs, whereas selected non-neuroendocrine or inflamed states can show higher MHC-I and inflammatory activity. The SCLC-I transcriptional state is enriched for immune-related signals (21), but this does not imply that every non-neuroendocrine tumor is inflamed or that every neuroendocrine-high tumor lacks immune infiltration. Direct measurement of MHC-I and immune context remains necessary.

Experimental studies further show that antigen-presentation repression is reversible in at least some SCLC states. Non-neuroendocrine, MHC-I-high tumor-cell states are more readily recognized by T cells and are associated with durable clinical benefit (25). In model systems, shifting away from neuroendocrine differentiation restored antigen presentation and innate immune signaling (25), Independent epigenetic perturbation restored MHC-I expression and MHC-I-restricted T-cell killing (45), while cytokine studies showed that HLA-I can remain inducible in selected HLA-low SCLC cells (46). These findings establish reversibility without implying that all MHC-I-low tumors share the same mechanism; the relevant epigenetic pathways are discussed in Section 5.

Whether the same lineage-immune relationship applies to SCLC transformed from EGFR-mutant lung adenocarcinoma remains unresolved. These tumors retain the founder EGFR alteration while acquiring neuroendocrine or SCLC-like features under targeted-therapy pressure (16, 17). Primary SCLC provides a mechanistic basis for hypothesizing that a neuroendocrine-high state may be accompanied by reduced MHC-I expression and weaker CD8^+^ T-cell recognition (21, 24, 25). However, paired longitudinal evidence showing that MHC-I loss, IFN suppression, or T-cell exclusion consistently emerges during transformation is limited. Immune invisibility in transformed SCLC should therefore be treated as a testable extrapolation rather than an established consequence of transformation.

But there are multiple ways this antigen presentation defect can come about, either by genetic or non-genetic means. Loss of HLA itself genetically or B2M disruption or other irreversible defects in antigen processing components prevents effective MHC-I display so makes checkpoint blockade less effective in these settings (38, 40, 41). However in many SCLC-like tumors they appear to actually suppress antigen presentation via reversible transcriptional or epigenetic mechanisms, and not via fixed genetic loss alone (25, 45, 46). Which is actually really important because it suggests you might not have lost anything forever genetically here if there was ever a chance of restoring antigen presentation, whereas true genetic loss of B2M or HLA would probably be very hard to restore back again.

Mechanistically, lineage-associated immune visibility may span a continuum from antigen-rich but presentation-defective tumors, through lineage-repressed states with reduced antigen presentation and inflammatory signaling, to more immune-visible non-neuroendocrine states. These represent mechanistic scenarios along a continuum rather than a separate tumor-immune classification framework (24, 25, 33, 34, 45, 46).

The above framework thus explains somewhat better why it seems usually insufficient when immune checkpoint blockade is applied alone against SCLC-like or lineage-transformed cancers. PD-1/PD-L1 blockers will help reviving any remaining anti-tumor T cells still around, but they won’t make up completely for missing antigen presentation. Without peptide-MHC-I complexes on the cell surface there may actually remain very little for CD8^+^ cells to target in the first place, no matter if checkpoints are closed or open. Hence, approaches which restore MHC-I expression, antigen processing and/or innate immune signaling may need to come before, or together with, checkpoint blockade in some cases of antigen-presentation repression; whereas where irreversible loss of antigen presentation has occurred then bypassing MHC-I altogether may become unavoidable, using things like T-cell engagers, antibody-drug conjugates etc.

In this review, impaired antigen presentation is considered a plausible bridge between lineage plasticity and immune invisibility rather than a uniform consequence of transformation. This relationship is supported most strongly by primary SCLC and experimental SCLC models, whereas paired clinical evidence before and after LUAD-to-SCLC transformation remains limited. Lineage state may influence whether tumor antigens are displayed and recognized by T cells, but the frequency, timing, and clinical relevance of these changes in transformed SCLC require direct longitudinal validation. Antigen-presentation defects should also be interpreted together with IFN signaling, chemokine production, immune-cell entry, and suppressive myeloid or stromal ecology.

## 4 Beyond MHC-I: IFN signaling, immune entry and suppressive myeloid ecology

But loss of antigen presentation is just one part of the whole story. There are many other factors that prevent antitumor responses from taking place against lineage-plastic lung cancer, even if some tumor cells express MHC-I. One reason is that CD8^+^ T cells need to get inside the tumor mass; they need to move close enough to target malignant cells; lastly, they need to stay activated and kill targets. Each requires different IFN signaling pathways. Chemokines attract effector T cells through chemokine receptors such as CXCR3 and CCR5, whereas stromal and vascular barriers can determine whether these cells reach malignant cell nests. Fibroblasts and extracellular matrix remodeling can either permit immune-cell migration or, more commonly in immune-excluded tumors, create stromal barriers that restrict T-cell access. Finally, some immune cells help activate others while others suppress their neighbors. So lineage-associated immune resistance shouldn’t be viewed merely as failure of tumor cell recognition. There may also be a problem of entry or local immune function in the tumor interior (47, 48).

IFN-γ signaling provides a critical link between tumor recognition and immune recruitment. In responsive tumors, IFN-γ produced by activated T cells and NK cells can induce antigen presentation genes, PD-L1 expression and chemokines that attract additional effector lymphocytes. A pan-tumor analysis showed that an IFN-γ-related mRNA profile was associated with clinical response to PD-1 blockade, supporting the importance of a pre-existing inflammatory circuit for checkpoint inhibitor sensitivity (49). Large-scale biomarker analyses have similarly suggested that T-cell-inflamed gene expression and tumor mutational burden provide complementary information for predicting response to PD-1 blockade-based therapy (11). Acquired JAK1 or JAK2 alterations have been established as mechanisms of resistance to PD-1 blockade in melanoma, providing a broader mechanistic precedent for how loss of IFN responsiveness can impair immune control (41). Although disruption of IFN signaling may also contribute to resistance in lung cancer, direct evidence for recurrent acquired JAK1/JAK2-mediated resistance in lineage-plastic lung tumors remains limited. These findings suggest that immune recognition requires not only antigen display, but also an inflammatory feedback loop that allows T cells to accumulate and function within the tumor.

The CXCL9/CXCL10/CXCL11-CXCR3 axis is one of the most important chemokine systems connecting IFN signaling to T-cell entry. CXCL9, CXCL10 and CXCL11 can recruit CXCR3-expressing effector T cells and NK cells into inflamed tissues, including tumors (50). In preclinical models and patient samples, macrophage-derived CXCL9 and CXCL10 were required for CD8^+^ T-cell infiltration and therapeutic efficacy after immune checkpoint blockade (51). This indicates that some myeloid cells can support antitumor immunity by producing T-cell-recruiting chemokines. However, the same myeloid compartment can also become immunosuppressive depending on tumor-derived signals, tissue context and treatment history.

Lineage plasticity may interfere with this immune-recruitment circuit at several levels. A tumor that shifts toward an SCLC-like or neuroendocrine-high state may reduce IFN-responsive gene expression, chemokine production and immune-cell-attracting signals, thereby creating an immune-excluded state even before strong T-cell dysfunction develops. Tumor cells can shape immune contexture through paracrine and juxtacrine communication with stromal and immune cells, making tumor-cell state a potential upstream contributor to the local immune ecology (52). Conversely, non-neuroendocrine or inflamed-like SCLC states may show stronger IFN and T-cell-related signals. The SCLC-I state described by Gay and colleagues is characterized by low expression of canonical lineage transcription factors and an inflamed immune signature, and it showed greater benefit from the addition of immunotherapy to chemotherapy (21). YAP1-associated SCLC has also been linked to a T-cell-inflamed phenotype, further supporting the idea that less classical neuroendocrine states can be more immune-engaged (22).

However, inflammation does not always mean effective antitumor immunity. Some tumors contain T-cell-related signals but remain poorly controlled because the infiltrating immune cells are spatially restricted, functionally impaired or dominated by suppressive myeloid cells. T-cell dysfunction in cancer is regulated by antigenic stimulation, inhibitory receptors, cytokines, metabolic stress and suppressive cells within the tumor microenvironment (53, 54). The TIDE framework separated T-cell dysfunction and T-cell exclusion as distinct barriers to immunotherapy response, which is conceptually useful for lineage-plastic tumors (55). In the IMpower133-based analysis by Nabet and colleagues, immune-inflamed SCLC subsets were not uniform. Neuroendocrine tumors with high T-effector signals and low tumor-associated macrophage signals appeared to derive greater benefit from atezolizumab plus chemotherapy, whereas non-neuroendocrine tumors with both high T-effector and high macrophage signals did not show the same advantage (23). This finding is important because it separates immune inflammation from immune effectiveness.

Spatial organization also matters. Single-cell profiling of human lung tumors has shown that the lung tumor microenvironment contains diverse immune, fibroblast and endothelial cell states that are molded by malignant tissue context (56). Spatial profiling of lung adenocarcinoma further showed that immune-cell composition and localization vary across histologic patterns and carry prognostic information (57). For lineage-plastic tumors, this raises a key question: does lineage switching only alter tumor-cell-intrinsic antigen presentation, or does it also reshape where immune cells are allowed to enter and interact with malignant cells? SCLC-like transformation may reduce immune-cell recruitment by weakening chemokine networks, while mixed or inflamed states may permit immune entry but still impose myeloid or stromal suppression.

The stromal compartment can further reinforce immune exclusion. Cancer-associated fibroblasts are heterogeneous stromal cells that regulate extracellular matrix remodeling, angiogenesis, therapy resistance and tumor immunity (58). In urothelial cancer, transforming growth factor-β signaling in fibroblast-rich tumors was associated with exclusion of T cells from the tumor parenchyma and resistance to PD-L1 blockade (59). In genetically reconstituted colon cancer metastasis models, TGF-β blockade increased T-cell infiltration and sensitized tumors to PD-1/PD-L1 inhibition, supporting a role for TGF-β-mediated stromal exclusion in that experimental setting (60). Whether the same mechanism contributes to immune resistance during LUAD-to-SCLC transformation remains unknown. Although these studies were not specific to lung cancer lineage plasticity, they provide a useful conceptual model: tumor cells may be antigenic and T cells may be present at the invasive margin, but stromal barriers can prevent productive tumor-cell contact. Evidence from melanoma, colorectal cancer, urothelial cancer, and other tumor types is included here as mechanistic precedent rather than as direct evidence for LUAD-to-SCLC transformation. These studies are relevant because HLA-I loss, IFN-pathway disruption, TGF-β-mediated stromal exclusion, and myeloid suppression regulate broadly conserved steps of antitumor immunity. However, their operation during SCLC transformation must be confirmed in paired pre- and post-transformation specimens, lineage-matched experimental models, and spatial or single-cell immune analyses.

Myeloid cells are therefore not simply background components of the tumor microenvironment. They can determine whether a lineage-shifted tumor becomes immune-inflamed, immune-excluded or immune-dysfunctional. Tumor-associated macrophages, monocytes, neutrophils and myeloid-derived suppressor cells can inhibit CD8^+^ T-cell function through PD-L1, arginase activity, reactive oxygen species, IL-10, TGF-β, prostaglandins, adenosine metabolism and other mechanisms (61–64). They can also affect antigen presentation indirectly by limiting dendritic-cell activation, reducing T-cell priming and creating metabolic competition within the tumor. In SCLC and SCLC-like tumors, the balance between T-effector signals and macrophage-associated suppression may be particularly important, as suggested by clinical transcriptomic analyses of immunotherapy-treated SCLC (23). Antigen-presentation loss also reshapes interactions between innate and adaptive immunity. Dendritic cells can acquire tumor-derived antigens and cross-present them to prime tumor-specific CD8^+^ T cells, but successful priming does not guarantee tumor-cell killing when malignant cells lack sufficient surface peptide-HLA-I complexes. Conversely, reduced HLA-I expression may relieve inhibitory KIR- or NKG2A-mediated signaling and increase susceptibility to NK-cell killing through missing-self recognition. However, HLA-I loss alone does not ensure effective NK-cell activity, which also depends on activating ligands, NK-cell infiltration, and the local suppressive microenvironment. Continued selection by T cells and NK cells may further drive immunoediting toward clones that combine defective antigen presentation with reduced NK-cell-activating signals or enhanced myeloid and stromal suppression. Lineage-associated immune invisibility should therefore be viewed as a dynamic interaction among tumor cells, dendritic cells, NK cells, and T cells rather than as an exclusively CD8^+^ T-cell-centered process (47, 48, 65–68).

Building on existing tumor-immune phenotype frameworks, we organize lineage-plastic lung cancers into three non-mutually exclusive practical states. Immune-invisible disease is characterized primarily by impaired MHC-I antigen processing and limited tumor-cell recognition. Immune-excluded disease may retain antigenicity but lacks effective immune-cell entry because of defective chemokine networks, vascular restriction, or stromal barriers. Immune-dysfunctional disease contains infiltrating lymphocytes that are locally suppressed by myeloid cells, regulatory T cells, fibroblasts, or inhibitory signaling. These states may coexist spatially or change during treatment. The specific contribution of this framework is not to propose a new universal classification, but to link immune phenotype with lineage transition, the reversibility of antigen-presentation defects, and the retention of actionable surface targets. This linkage may help distinguish tumors suited to immune restoration from those requiring immune redirection.

This framework helps explain why PD-1/PD-L1 blockade alone may be insufficient in lineage-plastic tumors. Checkpoint blockade has no substrate in immune-invisible tumors lacking enough antigen presenting capacity; T cells cannot get to cancer cells sufficiently well in immune-excluded tumors; while although T cells may actually be present they can still get shut off by myeloid or stromal cells in immune-dysfunctional tumors. Hence rational therapy requires matching what the dominant barrier is, restoring MHC-I and antigen processing probably matters most in immune-invisible cancers; enhancing IFN chemokine networks, vascular access or stromal permissiveness being needed for immune-excluded cancers; whereas targeting macrophages, myeloid suppression, Tregs or alternative checkpoints having greatest importance within immune-dysfunctional tumors.

Taken together, lineage-associated immune resistance extends beyond antigen presentation loss. The lineage state of tumor cells can shape IFN responsiveness, chemokine output, T-cell entry and the composition of the suppressive myeloid niche. These layers determine whether a tumor that is theoretically recognizable becomes practically visible to the immune system. The next section considers how epigenetic control of lineage state may stabilize these immune-invisible or immune-excluded states, and why epigenetic therapy may have value as an immune-restoring rather than purely cytotoxic strategy.

## 5 Epigenetic control of lineage state and immune visibility

Lineage plasticity is not only a transcriptional phenomenon, but also an epigenetically stabilized cell-state transition. When lung cancer cells acquire SCLC-like or neuroendocrine-high features, they do not simply turn on markers such as ASCL1, NEUROD1, INSM1, synaptophysin or chromogranin A. They also remodel chromatin accessibility, enhancer usage and repressive histone marks, thereby changing the expression of antigen presentation genes, IFN-responsive genes and immune-regulatory pathways. This is why epigenetic regulation is central to the concept of lineage-associated immune invisibility. It provides a mechanism through which a lineage state can become fixed as an immune-silent state (69).

Polycomb repressive complex 2 (PRC2), whose catalytic component EZH2 deposits H3K27me3, is one important link between developmental lineage control and immune evasion. A genome-wide CRISPR/Cas9 screen showed that PRC2 can silence the MHC-I antigen processing and presentation pathway and enable tumor cells to evade T-cell-mediated immunity (70). In MHC-I-low cancers, promoters of antigen presentation genes can carry bivalent chromatin features, including both H3K4me3 and H3K27me3, limiting basal expression and restricting cytokine-induced upregulation (70, 71). This finding is important for lineage-plastic tumors because it suggests that immune invisibility may be reversible when antigen presentation is suppressed by chromatin state rather than by irreversible genetic loss of HLA or B2M.

Regarding SCLC, EZH2 deserves special attention because neuroendocrine tumors may take advantage of developmentally regulated and stem-like chromatin patterns. Indeed, experimental research showed the presence of non-neuroendocrine MHC-I-high cell states in SCLC, which were more immunogenic and linked to longer survival following treatment with immune checkpoint inhibitors (25): actually, in that study EZH2 inhibition together with STING agonism led to the exposure of SCLC immunogenicity and contributed to T cell mediated killing of tumor cells (25), indicating that chromatin repression and innate immune signaling can decide whether SCLC becomes immune-visible or not. Moreover, EZH2 appears to play a key part also in acquired chemoresistance in SCLC through epigenetic inactivation of SLFN11 (72); hence, inhibition of EZH2 activity can be seen here as something that is not only supposed to bring about some sort of ‘cytostasis’ effect on cancer, but above all restore the capacity of the immune system to attack the neoplasm.

A final piece of evidence concerns LSD1 (KDM1A), which also shows connection between epigenetic therapy and lineage remodeling with immune recognition. LSD1 inhibition activates NOTCH signaling in SCLC while suppressing ASCL1 and reducing neuroendocrine lineage features (73). As ASCL1 is responsible for regulating neuroendocrine identity in this disease, LSD1 inhibition can therefore be regarded as one kind of drug treatment approach in order to force certain types of SCLC cells to move far away from their original classical neuroendocrine status. Actually, ASCL1 and NEUROD1 both represent two types of pulmonary neuroendocrine tumor status that regulate diverse kinds of transcriptional output respectively. So this indicates that the whole mechanism of maintaining SCLC lineage identity actually consists of some transcriptional activities together with the regulation of chromosome structure within each special state (74). Moreover, super-enhancer studies further prove that ASCL1, NKX2–1 and PROX1 could jointly influence subtype-specific gene expression in SCLC-A and reveal the association between lineage-decision transcription factors and chromatin organization (75). What is more interesting, however, when there exists weaker neuroendocrine activity, or even no neuroendocrine activity, then these kinds of cancers might appear much stronger in terms of displaying more obvious inflammatory reactions and expressing higher levels of antigens.

In another study carried out more directly with SCLC models, LSD1 inhibition had obvious immune consequences. Targeting LSD1 restored MHC-I surface expression, activated antigen-presentation genes and sensitized SCLC cells to MHC-I-restricted T-cell killing (45). Furthermore, it was found that Bomedemstat, which is an irreversible LSD1 inhibitor, enhanced the response to PD-1 blockade in a syngeneic SCLC model, increased CD8^+^ T-cell infiltration, increased MHC-I expression on tumor cells and augmented IFN-γ-induced MHC-I upregulation (76). Such results show clearly one strong example demonstrating immune restoration through lineage-state modulation because LSD1 inhibition here is not just applying more cytotoxic pressure but also modulating cell states to make tumors much easier to recognize by T-cells and more susceptible to T-cell killing.

Another important pathway which shows the interconnection between developmental fate-control pathways and neuroendocrine identity is the NOTCH-YAP-REST axis. First, NOTCH signaling itself seems able to create non-neuroendocrine tumor cell states inside of SCLC leading to intratumoral heterogeneity (31). Then there is evidence from mouse models showing that there exists a conserved YAP/Notch/REST network in the lung controlling decisions about whether to commit to neuroendocrine versus non-neuroendocrine fates (77). Thirdly, POU2F3-driven tuft-cell-like SCLC constitutes yet another case of a non-classical lineage state which turns out to have its basis in distinct transcriptional dependencies (30). Finally, immunohistochemistry on clinical SCLC samples has shown indeed that patients harbor different levels of ASCL1, NEUROD1, POU2F3 and YAP1, suggesting thus that these lineage states are relevant beyond cell-line models (29). Altogether then, such experiments point toward the conclusion that SCLC lineage identity isn’t fixed once and for all but rather is determined by reversible signaling circuits and transcriptional programs which may affect properties like tumor growth, immune phenotype and therapeutic vulnerability.

These findings also underscore the need for biomarker-guided application of epigenetic therapies. A lineage altering drug will probably behave differently according to what the original state of the cancer is. So NE-high, MHC-I-low tumors might benefit through inhibition of either EZH2 or LSD1 by regaining ability to present antigens again, becoming more active with regards to innate immune signaling, moving cells back away from a neuroendocrine state etc. But if instead we face irreversible B2M loss, HLA loss and profound immune exclusion, then such strategies won’t suffice since the whole antigen presenting pathway cannot ever come back properly again. Thus epigenetic-immuno combination therapy needs to take place together along with biomarker profiling of current lineage status, antigen presentation potential as well as IFN responsiveness.

Epigenetic regulation may differ between primary and transformed SCLC, although direct comparative evidence remains limited. Primary SCLC arises within a neuroendocrine developmental context and contains chromatin programs capable of repressing antigen-presentation and immune-response genes (20, 25, 45, 69). By contrast, transformed SCLC develops from LUAD under targeted-therapy pressure and may acquire only a subset of the lineage-associated chromatin features observed in primary SCLC (16, 17). Whether transformed SCLC represses MHC-I and IFN-responsive genes through the same epigenetic mechanisms as primary SCLC remains unknown. Direct comparisons of paired LUAD and post-transformation SCLC specimens will be required to determine whether acquisition of SCLC-like lineage programs is accompanied by corresponding changes in chromatin-mediated immune regulation.

Overall, epigenetic regulation can stabilize neuroendocrine-high, MHC-I-low, and IFN-low states while also creating a potential route to restore tumor recognition. The most important unresolved question is not whether epigenetic agents inhibit tumor growth, but whether they can reproducibly restore antigen presentation in biomarker-defined tumors without creating countervailing toxicity or suppressive inflammation. Their clinical value in lineage-plastic lung cancer remains investigational.

## 6 Therapeutic implications: restore visibility or redirect immunity

The therapeutic implication of lineage-associated immune resistance is that checkpoint blockade should not be treated as a universal solution. PD-1/PD-L1 inhibitors can release inhibitory signaling in responsive T cells, but they cannot compensate fully for absent antigen presentation, weak chemokine networks, or spatial exclusion. Clinically established strategies include atezolizumab or durvalumab with platinum-etoposide in extensive-stage SCLC (6, 7). and durvalumab after concurrent chemoradiotherapy in limited-stage SCLC (8). By contrast, epigenetic combinations, STING agonists, most antigen-directed ADCs, and cellular therapies remain preclinical or early clinical. Many patients still derive limited or non-durable benefit, and the immune responsiveness of transformed SCLC remains poorly defined (24, 26). **Table 1** summarizes the evidence levels supporting the major therapeutic approaches.

**Table 1:** Evidence map for lineage-associated immune resistance in lung cancer.

Evidence domain	Clinical or biological setting	Key immune finding	Clinical or therapeutic implication	Evidence basis
ICI benefit	Advanced NSCLC and SCLC	Clinical benefit is restricted to selected immune contexts	Use ICIs according to established disease-specific indications	Phase III randomized trials
Oncogene-driven resistance	EGFR-mutant or ALK-rearranged NSCLC	Low response rates and limited T-cell inflammation	ICI monotherapy generally has limited activity	Retrospective clinical cohorts
SCLC transformation	EGFR-mutant LUAD after EGFR-TKI treatment	Immune consequences remain inferential; paired longitudinal evidence is limited	Consider tissue re-biopsy and prospective immune profiling when transformation is suspected	Paired-biopsy and retrospective clinical studies
Transformation risk	EGFR/TP53/RB1-altered LUAD	Genomic background is associated with increased risk of SCLC transformation	Closer clinical and pathological monitoring may be warranted	Retrospective clinical-genomic cohorts
Transcriptional subtype	Primary SCLC	ASCL1-, NEUROD1-, POU2F3-, and inflamed-associated states show variable immune activity	Transcriptional subtype alone is insufficient for immune-based treatment selection	Clinical transcriptomic classifications
Immune heterogeneity	Primary SCLC	The balance between T-effector and macrophage-associated signals may influence ICI benefit	Integrate lineage state with immune context	Trial-associated translational analyses
Antigen-presentation capacity	CheckMate 032 SCLC specimens	Higher APM/MHC-I activity is associated with durable clinical benefit	Assess antigen-presentation capacity in addition to transcriptional subtype	Clinical correlative analysis
MHC-I-high non-NE state	Non-neuroendocrine SCLC states	Increased MHC-I expression and T-cell recognition occur in selected plastic states	Supports the potential reversibility of immune invisibility in selected tumors	Preclinical evidence with clinical association
Epigenetic regulation	SCLC models	EZH2- or LSD1-associated repression of antigen-presentation genes may be reversible	Provides a rationale for investigational immune-restoration strategies	Preclinical mechanistic evidence
DLL3-directed T-cell engagement	Previously treated SCLC	T-cell engagement is independent of endogenous peptide-MHC-I recognition; clinical trials were not stratified by MHC-I/HLA-I status	Clinically active in previously treated SCLC, but the predictive value of MHC-I status remains unknown	Phase II and phase III trials without MHC-I-based patient selection
Other surface-targeted strategies	SCLC models and early clinical studies	B7-H3, TROP2, SEZ6, or DLL3 can support targeting independently of endogenous peptide-MHC-I recognition	ADC and CAR-T/CAR-NK approaches remain investigational	Preclinical to early clinical evidence

ADC, antibody-drug conjugate; APM, antigen-processing machinery; CAR, chimeric antigen receptor; ICI, immune checkpoint inhibitor; LUAD, lung adenocarcinoma; NSCLC, non-small-cell lung cancer; SCLC, small-cell lung cancer; TKI, tyrosine kinase inhibitor. Evidence categories describe the principal study design supporting each conclusion and do not constitute a formal evidence-grading system.

**Figure 3:**
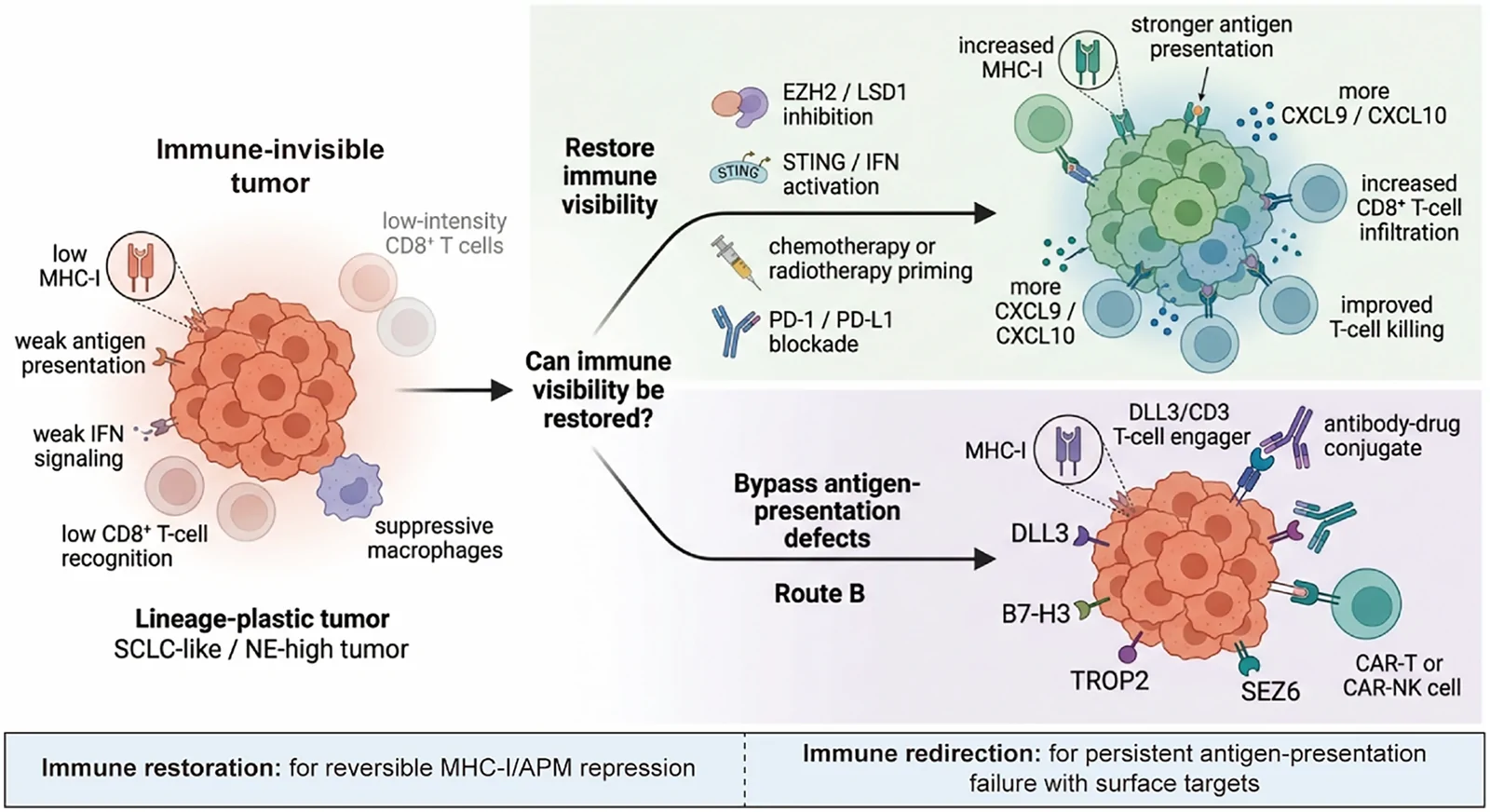
Therapeutic strategies for distinct forms of lineage-associated immune resistance. Tumors with reversible repression of MHC-I or antigen-processing genes may be candidates for immune-restoration strategies that recover endogenous recognition. EZH2 or LSD1 inhibition and STING/IFN activation are supported mainly by preclinical evidence, whereas chemotherapy, radiotherapy, and checkpoint blockade have established roles in defined clinical settings. Tumors with persistent antigen-presentation defects but retained surface targets may instead be approached through immune redirection. DLL3-directed T-cell engagement is supported by phase II and phase III clinical evidence in previously treated SCLC, although these studies did not select or stratify patients according to MHC-I or HLA-I status. Its clinical activity therefore supports DLL3-directed immune redirection but does not establish preferential efficacy in MHC-I-deficient tumors. Most ADC and CAR-T/CAR-NK strategies remain investigational. Treatment selection should consider both the dominant immune barrier and the level of evidence supporting each intervention. Created in BioRender. https://BioRender.com/rqfpvjx.

A practical way to organize future treatment is to ask whether the tumor should be made visible again or whether immune recognition must be redirected around classical antigen presentation. Immune restoration aims to recover tumor-cell recognition by increasing MHC-I expression, antigen processing, IFN signaling, chemokine production and T-cell entry. Immune redirection aims to bypass defective MHC-I-restricted recognition by using lineage-associated surface targets, bispecific T-cell engagers, antibody-drug conjugates or cellular therapies. These two strategies are not mutually exclusive, but they are suited to different immune states. These therapeutic directions are illustrated in **Figure 3**.

### 6.1 Immune restoration: making lineage-plastic tumors visible again

Immune restoration is most relevant when antigen presentation is suppressed but potentially reversible. This may apply to tumors with transcriptional or epigenetic repression of MHC-I, TAP1, TAP2, B2M or IFN-responsive genes, rather than irreversible genetic loss of HLA or B2M. In this setting, the goal is not simply to add another cytotoxic agent, but to re-establish the conditions required for CD8^+^ T-cell recognition and checkpoint blockade sensitivity (38, 40).

Preclinical studies support epigenetic restoration of antigen presentation. PRC2/EZH2 can silence the MHC-I pathway (70), and combined EZH2 inhibition and STING activation exposed latent immunogenicity in SCLC models (25). LSD1 inhibition restored MHC-I and sensitized SCLC cells to MHC-I-restricted T-cell killing (45), while bomedemstat enhanced MHC-I expression, CD8^+^ T-cell infiltration, and PD-1 blockade activity in a syngeneic model (76). These findings provide a mechanistic rationale for clinical investigation but do not yet establish epigenetic immune restoration as a standard treatment.

Innate immune activation is another investigational restoration strategy. The cGAS-STING pathway links cytosolic DNA sensing to type I IFN production, dendritic-cell activation, and CD8^+^ T-cell priming (65, 66). Radiotherapy can generate cytosolic DNA and STING-dependent IFN signaling under appropriate dose and fractionation conditions (78). However, STING activation can also promote suppressive inflammation and myeloid-cell recruitment (79). Biomarker selection and treatment context will therefore be essential before STING-based combinations can be incorporated into routine clinical practice.

Chemotherapy and radiotherapy may also contribute to restoration by releasing antigens, increasing inflammatory signaling and altering tumor-immune interactions. This may partly explain why PD-L1 blockade has been most successful in SCLC when added to chemotherapy rather than used as single-agent therapy in unselected patients (6, 7). The key point is that these treatments may create or amplify an immune substrate for checkpoint blockade. For lineage-plastic tumors with low antigen presentation, the most rational combinations are those that restore MHC-I, strengthen IFN-responsive chemokines and improve T-cell access before expecting PD-1/PD-L1 blockade to work.

Immune restoration also requires attention to the suppressive microenvironment. If a tumor is MHC-I-positive but dominated by macrophages, myeloid-derived suppressor cells, Tregs or stromal exclusion, then restoring antigen presentation alone may be insufficient. In SCLC, clinical trial-based transcriptomic analysis showed that the balance between T-effector signals and macrophage-associated signals may influence benefit from PD-L1 blockade plus chemotherapy (23). Therefore, restoration strategies may need to combine antigen-presentation enhancement with myeloid reprogramming, T-cell recruitment or stromal remodeling (55, 62).

### 6.2 Immune redirection: bypassing defective antigen presentation

When MHC-I antigen presentation is profoundly impaired or genetically lost, immune restoration may not be enough. In that context, immune redirection becomes more attractive. This approach uses cell-surface targets to bring immune effector mechanisms to tumor cells without relying entirely on endogenous peptide-MHC-I recognition. For SCLC-like and neuroendocrine-high tumors, DLL3 is the most developed example of a lineage-associated surface target. DLL3 is highly expressed in SCLC and other high-grade neuroendocrine tumors but has limited expression in most normal tissues, making it an attractive target for antibody-based therapy (80, 81).

The early clinical development of the DLL3-targeted ADC rovalpituzumab tesirine established DLL3 as a druggable surface target in recurrent SCLC (82). However, the phase III TAHOE trial subsequently showed inferior overall survival and greater toxicity with rovalpituzumab tesirine than with topotecan, demonstrating that the validity of a lineage-associated target does not ensure the success of a particular ADC design (83). By contrast, tarlatamab, a DLL3/CD3 bispecific T-cell engager, redirects T cells toward DLL3-positive tumor cells without requiring endogenous peptide-MHC-I recognition. It showed clinically meaningful activity in the phase II DeLLphi-301 study (84) and improved overall survival compared with standard second-line chemotherapy in the phase III DeLLphi-304 trial (85). However, these trials did not select or stratify patients according to tumor MHC-I or HLA-I status. Their results therefore validate DLL3-directed T-cell engagement as an effective therapeutic strategy in previously treated SCLC, but do not establish preferential efficacy in MHC-I-deficient tumors. Prospective biomarker analyses are required to determine whether antigen-presentation status influences clinical benefit from DLL3-directed therapy.

The clinical activity of DLL3-directed T-cell engagers must nevertheless be balanced against their toxicity and treatment burden. Cytokine release syndrome and neurologic adverse events are clinically relevant, particularly during early treatment, and step-up dosing with close observation is used to mitigate these risks (84, 85). These requirements may increase the logistical burden of treatment and restrict accessibility outside centers experienced in managing T-cell-engaging therapies. Such considerations are important when immune-redirection strategies are compared with approaches intended to restore endogenous antitumor immunity.

Beyond DLL3-directed T-cell engagement, ADCs against B7-H3, TROP2, SEZ6 and other surface targets are being developed in SCLC (86). B7-H3-directed ADCs have advanced into clinical investigation (86, 87), whereas the SEZ6-directed ADC ABBV-011 has shown preclinical activity in SCLC models (88). These approaches reinforce the potential value of surface-antigen-based treatment when endogenous antigen presentation is impaired. However, the extent to which expression of B7-H3, TROP2 or SEZ6 is determined by lineage state, rather than by broader tumor heterogeneity, remains less clearly defined.

Cellular therapies also fit mechanistically within the immune-redirection framework. Unlike endogenous T-cell receptors, chimeric antigen receptors recognize intact cell-surface antigens directly and can mediate cytotoxicity independently of MHC restriction (67). CAR-T or CAR-NK cells targeting DLL3, B7-H3 or other neuroendocrine-associated surface antigens could therefore retain tumor-recognition capacity in MHC-I-deficient disease, provided that the selected surface antigen remains sufficiently and selectively expressed. Their target recognition does not require tumor PD-L1 expression, although PD-1/PD-L1 signaling and other suppressive pathways may still impair effector-cell function (67).

CAR-NK therapy may offer an additional, although conditional, missing-self advantage. HLA-I molecules normally restrain NK-cell activation through inhibitory receptors such as KIRs and CD94/NKG2A. Consequently, HLA-I loss that impairs CD8^+^ T-cell recognition may simultaneously reduce inhibitory signaling to NK cells and increase susceptibility to NK-cell-mediated killing (67, 68). CAR-NK cells could potentially combine this endogenous missing-self recognition with CAR-mediated targeting of a retained lineage-associated surface antigen.

However, MHC-I loss alone does not ensure effective CAR-NK activity, and substantial barriers remain for both CAR-T and CAR-NK therapy in solid tumors. These include heterogeneous or unstable target expression, antigen escape, on-target/off-tumor toxicity, limited trafficking into tumor tissue, insufficient persistence, cellular exhaustion and suppression by myeloid, stromal and metabolic components of the tumor microenvironment (67). Clinical application will therefore require assessment of target density, intratumoral heterogeneity and spatial immune context rather than histologic diagnosis alone.

### 6.3 Toward lineage-informed immune stratification

A lineage-informed assessment should integrate multiple biological layers rather than rely on histology, driver-mutation status or PD-L1 expression alone. The first layer is lineage state, with particular attention to LUAD-like, SCLC-like/neuroendocrine-high, and non-neuroendocrine or inflamed-like states; squamous-like or mixed states should be documented when present but should not be assumed to share the same immune biology. The second layer is antigen-presentation status, including MHC-I, B2M, TAP1, TAP2, HLA loss of heterozygosity and IFN-inducible antigen-presentation capacity. The third is immune-entry status, including IFN signaling, CXCL9/CXCL10/CCL5 expression, vascular accessibility and T-cell localization. The fourth is the suppressive immune ecology, including macrophages, monocytes, neutrophils, MDSCs, Tregs, CAFs and inhibitory checkpoints. The fifth is surface-target status, including DLL3, B7-H3, TROP2 and other potentially actionable lineage-associated antigens.

This framework can help match therapy to the dominant immune barrier. Immune-invisible tumors with reversible MHC-I repression may be candidates for epigenetic therapy, STING/IFN activation, radiotherapy or chemotherapy combined with immune checkpoint blockade. Immune-excluded tumors may require restoration of chemokine networks, vascular access or stromal permissiveness. Immune-dysfunctional tumors may require myeloid or Treg-directed combinations. Tumors with irreversible antigen-presentation loss but strong surface-target expression may be better suited for T-cell engagers, antibody-drug conjugates or cellular therapy.

Implementing this framework requires consideration of both temporal evolution and spatial sampling error. A single core biopsy may overrepresent one tumor region and fail to capture coexisting neuroendocrine, non-neuroendocrine, MHC-I-high, MHC-I-low, inflamed or myeloid-dominated areas, consistent with the spatial heterogeneity of HLA expression and immune-cell localization documented in lung cancer (42, 56, 57). Repeat biopsy at progression may therefore be informative when histologic transformation is suspected, particularly because lineage conversion may be missed by plasma-based genotyping alone (15–17, 27). However, repeat tissue sampling may be limited by lesion accessibility, procedural risk, tissue quality and the clinical condition of the patient.

A pragmatic near-term approach may begin with conventional histopathology and a focused immunohistochemical panel applied to available diagnostic or progression tissue, including neuroendocrine markers, MHC-I/B2M, DLL3 and selected immune markers. ctDNA may assist in tracking genomic evolution and persistence of founder driver alterations, but it cannot define histologic transformation or resolve spatial immune architecture (27). Single-cell sequencing and spatial proteomic or transcriptomic methods may provide more comprehensive information (56, 57), but their routine implementation remains limited by tissue requirements, cost, turnaround time, analytical standardization and the absence of prospectively validated decision thresholds. The proposed framework should therefore be viewed as a tiered strategy rather than a requirement that every patient undergo all available assays.

### 6.4 Limitations and outstanding questions

Several limitations of the proposed framework should be acknowledged. First, primary and transformed SCLC are not biologically interchangeable because they differ in cellular origin, treatment history, founder genotype, and evolutionary context. Evidence derived from these tumors, oncogene-driven NSCLC, clinical correlative datasets, and experimental models therefore cannot be combined without qualification. Paired longitudinal specimens collected before and after SCLC transformation remain uncommon, and direct evidence that antigen-presentation loss consistently accompanies transformation is limited (15–17). Second, the immune consequences of squamous-like, mixed, and other non-neuroendocrine transitions are less well established because available studies have focused mainly on histologic evolution and treatment resistance (27, 28). Third, immune invisibility, immune exclusion, and T-cell dysfunction may coexist within different regions of the same tumor or change during treatment (23, 42, 57), making single-biopsy classification vulnerable to spatial sampling error. Fourth, no prospectively validated biomarker panel reliably distinguishes reversible transcriptional or epigenetic repression from irreversible HLA or B2M loss. Finally, neither the proposed stratification framework nor the distinction between immune restoration and immune redirection has been prospectively validated for treatment selection. These categories should therefore be regarded as an organizing model for future investigation rather than an established clinical classification.

## 7 Conclusions

Neuroendocrine lineage plasticity may provide a tumor-cell-intrinsic route to immune resistance in lung cancer, with the strongest evidence arising from primary SCLC and experimental SCLC models. During EGFR-mutant lung-adenocarcinoma-to-SCLC transformation, corresponding changes in antigen presentation and immune visibility remain biologically plausible but require confirmation in paired longitudinal specimens. The central therapeutic question is not simply whether PD-1/PD-L1 blockade should be added, but whether tumor cells remain recognizable, whether effector T cells can enter and function, and whether retained surface targets can be exploited when classical antigen presentation fails. Future strategies should distinguish clinically established treatments from investigational combinations and select immune restoration or redirection according to lineage state, antigen-presentation integrity, immune localization, and suppressive context.

## References

[1] Reck M Rodriguez-Abreu D Robinson AG Hui R Csoszi T Fulop A . Pembrolizumab versus chemotherapy for PD-L1-positive non-small-cell lung cancer. N Engl J Med. (2016) 375:1823–33. doi: 10.1056/nejmoa1606774 27718847

[2] Gandhi L Rodriguez-Abreu D Gadgeel S Esteban E Felip E De Angelis F . Pembrolizumab plus chemotherapy in metastatic non-small-cell lung cancer. N Engl J Med. (2018) 378:2078–92. doi: 10.1056/nejmoa1801005 29658856

[3] Paz-Ares L Luft A Vicente D Tafreshi A Gumus M Mazieres J . Pembrolizumab plus chemotherapy for squamous non-small-cell lung cancer. N Engl J Med. (2018) 379:2040–51. doi: 10.1056/nejmoa1810865 30280635

[4] Hellmann MD Paz-Ares L Bernabe Caro R Zurawski B Kim SW Carcereny Costa E . Nivolumab plus ipilimumab in advanced non-small-cell lung cancer. N Engl J Med. (2019) 381:2020–31. doi: 10.1056/nejmoa1910231 31562796

[5] Antonia SJ Villegas A Daniel D Vicente D Murakami S Hui R . Durvalumab after chemoradiotherapy in stage III non-small-cell lung cancer. N Engl J Med. (2017) 377:1919–29. doi: 10.1056/nejmoa1709937 28885881

[6] Horn L Mansfield AS Szczesna A Havel L Krzakowski M Hochmair MJ . First-line atezolizumab plus chemotherapy in extensive-stage small-cell lung cancer. N Engl J Med. (2018) 379:2220–9. doi: 10.1056/nejmoa1809064 30280641

[7] Paz-Ares L Dvorkin M Chen Y Reinmuth N Hotta K Trukhin D . Durvalumab plus platinum-etoposide versus platinum-etoposide in first-line treatment of extensive-stage small-cell lung cancer (CASPIAN): a randomised, controlled, open-label, phase 3 trial. Lancet. (2019) 394:1929–39. doi: 10.1016/s0140-6736(19)32222-6 31590988

[8] Cheng Y Spigel DR Cho BC Laktionov KK Fang J Chen Y . Durvalumab after chemoradiotherapy in limited-stage small-cell lung cancer. N Engl J Med. (2024) 391:1313–27. doi: 10.1056/nejmoa2404873 39268857

[9] Gainor JF Shaw AT Sequist LV Fu X Azzoli CG Piotrowska Z . EGFR mutations and ALK rearrangements are associated with low response rates to PD-1 pathway blockade in non-small cell lung cancer: a retrospective analysis. Clin Cancer Res Off J Am Assoc For Cancer Res. (2016) 22:4585–93. doi: 10.1158/1078-0432.ccr-15-3101 27225694 PMC5026567

[10] Rizvi NA Hellmann MD Snyder A Kvistborg P Makarov V Havel JJ . Cancer immunology. Mutational landscape determines sensitivity to PD-1 blockade in non-small cell lung cancer. Science. (2015) 348:124–8. doi: 10.1126/science.aaa1348 25765070 PMC4993154

[11] Cristescu R Mogg R Ayers M Albright A Murphy E Yearley J . Pan-tumor genomic biomarkers for PD-1 checkpoint blockade-based immunotherapy. Science. (2018) 362(6411):eaar3593. doi: 10.1126/science.aar3593 30309915 PMC6718162

[12] Ott PA Bang YJ Piha-Paul SA Razak ARA Bennouna J Soria JC . T-cell-inflamed gene-expression profile, programmed death ligand 1 expression, and tumor mutational burden predict efficacy in patients treated with pembrolizumab across 20 cancers: KEYNOTE-028. J Clin Oncol. (2019) 37:318–27. doi: 10.1200/jco.2018.78.2276 30557521

[13] Quintanal-Villalonga A Chan JM Yu HA Pe'er D Sawyers CL Sen T . Lineage plasticity in cancer: a shared pathway of therapeutic resistance. Nat Rev Clin Oncol. (2020) 17:360–71. doi: 10.1038/s41571-020-0340-z 32152485 PMC7397755

[14] Li X Gardner EE Molina-Pinelo S Wilhelm C Mu P Quintanal-Villalonga A . Lineage plasticity and histological transformation: tumor histology as a spectrum. Cell Res. (2025) 35:803–23. doi: 10.1038/s41422-025-01180-x 41023204 PMC12589604

[15] Sequist LV Waltman BA Dias-Santagata D Digumarthy S Turke AB Fidias P . Genotypic and histological evolution of lung cancers acquiring resistance to EGFR inhibitors. Sci Transl Med. (2011) 3:75ra26. doi: 10.1126/scitranslmed.3002003 21430269 PMC3132801

[16] Niederst MJ Sequist LV Poirier JT Mermel CH Lockerman EL Garcia AR . RB loss in resistant EGFR mutant lung adenocarcinomas that transform to small-cell lung cancer. Nat Commun. (2015) 6:6377. doi: 10.1038/ncomms7377 25758528 PMC4357281

[17] Marcoux N Gettinger SN O'Kane G Arbour KC Neal JW Husain H . EGFR-mutant adenocarcinomas that transform to small-cell lung cancer and other neuroendocrine carcinomas: clinical outcomes. J Clin Oncol. (2019) 37:278–85. doi: 10.1200/jco.18.01585 30550363 PMC7001776

[18] Offin M Chan JM Tenet M Rizvi HA Shen R Riely GJ . Concurrent RB1 and TP53 alterations define a subset of EGFR-mutant lung cancers at risk for histologic transformation and inferior clinical outcomes. J Thorac Oncol. (2019) 14:1784–93. doi: 10.1016/j.jtho.2019.06.002 31228622 PMC6764905

[19] Gardner EE Earlie EM Li K Thomas J Hubisz MJ Stein BD . Lineage-specific intolerance to oncogenic drivers restricts histological transformation. Science. (2024) 383:eadj1415. doi: 10.1126/science.adj1415 38330136 PMC11155264

[20] George J Lim JS Jang SJ Cun Y Ozretic L Kong G . Comprehensive genomic profiles of small cell lung cancer. Nature. (2015) 524:47–53. doi: 10.1038/nature14664 26168399 PMC4861069

[21] Gay CM Stewart CA Park EM Diao L Groves SM Heeke S . Patterns of transcription factor programs and immune pathway activation define four major subtypes of SCLC with distinct therapeutic vulnerabilities. Cancer Cell. (2021) 39:346–60:e7. doi: 10.1016/j.ccell.2020.12.014 33482121 PMC8143037

[22] Owonikoko TK Dwivedi B Chen Z Zhang C Barwick B Ernani V . YAP1 expression in SCLC defines a distinct subtype with T-cell-inflamed phenotype. J Thorac Oncol. (2021) 16:464–76. doi: 10.1016/j.jtho.2020.11.006 33248321 PMC7920957

[23] Nabet BY Hamidi H Lee MC Banchereau R Morris S Adler L . Immune heterogeneity in small-cell lung cancer and vulnerability to immune checkpoint blockade. Cancer Cell. (2024) 42:429–43:e4. doi: 10.1016/j.ccell.2024.01.010 38366589

[24] Rudin CM Balli D Lai WV Richards AL Nguyen E Egger JV . Clinical benefit from immunotherapy in patients with SCLC is associated with tumor capacity for antigen presentation. J Thorac Oncol. (2023) 18:1222–32. doi: 10.1016/j.jtho.2023.05.008 37210008 PMC10524620

[25] Mahadevan NR Knelson EH Wolff JO Vajdi A Saigi M Campisi M . Intrinsic immunogenicity of small cell lung carcinoma revealed by its cellular plasticity. Cancer Discov. (2021) 11:1952–69. doi: 10.1158/2159-8290.cd-20-0913 33707236 PMC8338750

[26] Simpson KL Rothwell DG Blackhall F Dive C . Challenges of small cell lung cancer heterogeneity and phenotypic plasticity. Nat Rev Cancer. (2025) 25:447–62. doi: 10.1038/s41568-025-00803-0 40211072

[27] Schoenfeld AJ Chan JM Kubota D Sato H Rizvi H Daneshbod Y . Tumor analyses reveal squamous transformation and off-target alterations as early resistance mechanisms to first-line osimertinib in EGFR-mutant lung cancer. Clin Cancer Res Off J Am Assoc For Cancer Res. (2020) 26:2654–63. doi: 10.1158/1078-0432.ccr-19-3563 31911548 PMC7448565

[28] Pathak R Villaflor VM . Histologic transformation in EGFR-mutant lung adenocarcinomas: mechanisms and therapeutic implications. Cancers (Basel). (2021) 13(18):4641. doi: 10.3390/cancers13184641 34572868 PMC8470834

[29] Baine MK Hsieh MS Lai WV Egger JV Jungbluth AA Daneshbod Y . SCLC subtypes defined by ASCL1, NEUROD1, POU2F3, and YAP1: a comprehensive immunohistochemical and histopathologic characterization. J Thorac Oncol. (2020) 15:1823–35. doi: 10.1016/j.jtho.2020.09.009 33011388 PMC8362797

[30] Huang YH Klingbeil O He XY Wu XS Arun G Lu B . POU2F3 is a master regulator of a tuft cell-like variant of small cell lung cancer. Genes Dev. (2018) 32:915–28. doi: 10.1101/gad.314815.118 29945888 PMC6075037

[31] Lim JS Ibaseta A Fischer MM Cancilla B O'Young G Cristea S . Intratumoural heterogeneity generated by Notch signalling promotes small-cell lung cancer. Nature. (2017) 545:360–4. doi: 10.1038/nature22323 28489825 PMC5776014

[32] Ireland AS Xie DA Hawgood SB Barbier MW Zuo LY Hanna BE . Basal cell of origin resolves neuroendocrine-tuft lineage plasticity in cancer. Nature. (2025) 647:257–67. doi: 10.1038/s41586-025-09503-z 40963028 PMC12589105

[33] Leone P Shin EC Perosa F Vacca A Dammacco F Racanelli V . MHC class I antigen processing and presenting machinery: organization, function, and defects in tumor cells. J Natl Cancer Inst. (2013) 105:1172–87. doi: 10.1093/jnci/djt184 23852952

[34] Jhunjhunwala S Hammer C Delamarre L . Antigen presentation in cancer: insights into tumour immunogenicity and immune evasion. Nat Rev Cancer. (2021) 21:298–312. doi: 10.1038/s41568-021-00339-z 33750922

[35] DhatChinamoorthy K Colbert JD Rock KL . Cancer immune evasion through loss of MHC class I antigen presentation. Front Immunol. (2021) 12:636568. doi: 10.3389/fimmu.2021.636568 33767702 PMC7986854

[36] Kallingal A Olszewski M Maciejewska N Brankiewicz W Baginski M . Cancer immune escape: the role of antigen presentation machinery. J Cancer Res Clin Oncol. (2023) 149:8131–41. doi: 10.1007/s00432-023-04737-8 37031434 PMC10374767

[37] McGranahan N Furness AJ Rosenthal R Ramskov S Lyngaa R Saini SK . Clonal neoantigens elicit T cell immunoreactivity and sensitivity to immune checkpoint blockade. Science. (2016) 351:1463–9. doi: 10.1126/science.aaf1490 26940869 PMC4984254

[38] McGranahan N Rosenthal R Hiley CT Rowan AJ Watkins TBK Wilson GA . Allele-specific HLA loss and immune escape in lung cancer evolution. Cell. (2017) 171:1259–71:e11. doi: 10.1158/1557-3265.aacriaslc18-ia23 29107330 PMC5720478

[39] So T Takenoyama M Mizukami M Ichiki Y Sugaya M Hanagiri T . Haplotype loss of HLA class I antigen as an escape mechanism from immune attack in lung cancer. Cancer Res. (2005) 65:5945–52. doi: 10.1158/0008-5472.can-04-3787 15994973

[40] Gettinger S Choi J Hastings K Truini A Datar I Sowell R . Impaired HLA class I antigen processing and presentation as a mechanism of acquired resistance to immune checkpoint inhibitors in lung cancer. Cancer Discov. (2017) 7:1420–35. doi: 10.1158/2159-8290.cd-17-0593 29025772 PMC5718941

[41] Zaretsky JM Garcia-Diaz A Shin DS Escuin-Ordinas H Hugo W Hu-Lieskovan S . Mutations associated with acquired resistance to PD-1 blockade in melanoma. N Engl J Med. (2016) 375:819–29. doi: 10.1056/nejmoa1604958 27433843 PMC5007206

[42] Datar IJ Hauc SC Desai S Gianino N Henick B Liu Y . Spatial analysis and clinical significance of HLA class-I and class-II subunit expression in non-small cell lung cancer. Clin Cancer Res Off J Am Assoc For Cancer Res. (2021) 27:2837–47. doi: 10.1158/1078-0432.ccr-20-3655 33602682 PMC8734284

[43] Tay RY Heigener D Reck M Califano R . Immune checkpoint blockade in small cell lung cancer. Lung Cancer. (2019) 137:31–7. doi: 10.1016/j.lungcan.2019.08.024 31525648

[44] Tanio Y Watanabe M Osaki T Tachibana I Kawase I Kuritani T . High sensitivity to peripheral blood lymphocytes and low HLA-class I antigen expression of small cell lung cancer cell lines with diverse chemo-radiosensitivity. Jpn J Cancer Res. (1992) 83:736–45. doi: 10.1111/j.1349-7006.1992.tb01974.x 1325431 PMC5918924

[45] Nguyen EM Taniguchi H Chan JM Zhan YA Chen X Qiu J . Targeting lysine-specific demethylase 1 rescues major histocompatibility complex class I antigen presentation and overcomes programmed death-ligand 1 blockade resistance in SCLC. J Thorac Oncol. (2022) 17:1014–31. doi: 10.1016/j.jtho.2022.05.014 35691495 PMC9357096

[46] Carbotti G Nikpoor AR Vacca P Gangemi R Giordano C Campelli F . IL-27 mediates HLA class I up-regulation, which can be inhibited by the IL-6 pathway, in HLA-deficient small cell lung cancer cells. J Exp Clin Cancer Res. (2017) 36:140. doi: 10.1186/s13046-017-0608-z 29020964 PMC5637329

[47] Binnewies M Roberts EW Kersten K Chan V Fearon DF Merad M . Understanding the tumor immune microenvironment (TIME) for effective therapy. Nat Med. (2018) 24:541–50. doi: 10.1038/s41591-018-0014-x 29686425 PMC5998822

[48] Roerden M Spranger S . Cancer immune evasion, immunoediting and intratumour heterogeneity. Nat Rev Immunol. (2025) 25:353–69. doi: 10.1038/s41577-024-01111-8 39748116

[49] Ayers M Lunceford J Nebozhyn M Murphy E Loboda A Kaufman DR . IFN-gamma-related mRNA profile predicts clinical response to PD-1 blockade. J Clin Invest. (2017) 127:2930–40. doi: 10.1172/jci91190 28650338 PMC5531419

[50] Tokunaga R Zhang W Naseem M Puccini A Berger MD Soni S . CXCL9, CXCL10, CXCL11/CXCR3 axis for immune activation - a target for novel cancer therapy. Cancer Treat Rev. (2018) 63:40–7. doi: 10.1016/j.ctrv.2017.11.007 29207310 PMC5801162

[51] House IG Savas P Lai J Chen AXY Oliver AJ Teo ZL . Macrophage-derived CXCL9 and CXCL10 are required for antitumor immune responses following immune checkpoint blockade. Clin Cancer Res Off J Am Assoc For Cancer Res. (2020) 26:487–504. doi: 10.1158/1078-0432.ccr-19-1868 31636098

[52] van Weverwijk A de Visser KE . Mechanisms driving the immunoregulatory function of cancer cells. Nat Rev Cancer. (2023) 23:193–215. doi: 10.1038/s41568-022-00544-4 36717668

[53] Speiser DE Ho PC Verdeil G . Regulatory circuits of T cell function in cancer. Nat Rev Immunol. (2016) 16:599–611. doi: 10.1038/nri.2016.80 27526640

[54] Hanahan D Michielin O Pittet MJ . Convergent inducers and effectors of T cell paralysis in the tumour microenvironment. Nat Rev Cancer. (2025) 25:41–58. doi: 10.1038/s41568-024-00761-z 39448877

[55] Jiang P Gu S Pan D Fu J Sahu A Hu X . Signatures of T cell dysfunction and exclusion predict cancer immunotherapy response. Nat Med. (2018) 24:1550–8. doi: 10.1038/s41591-018-0136-1 30127393 PMC6487502

[56] Lambrechts D Wauters E Boeckx B Aibar S Nittner D Burton O . Phenotype molding of stromal cells in the lung tumor microenvironment. Nat Med. (2018) 24:1277–89. doi: 10.1038/s41591-018-0096-5 29988129

[57] Sorin M Rezanejad M Karimi E Fiset B Desharnais L Perus LJM . Single-cell spatial landscapes of the lung tumour immune microenvironment. Nature. (2023) 614:548–54. doi: 10.1038/s41586-022-05672-3 36725934 PMC9931585

[58] Kalluri R . The biology and function of fibroblasts in cancer. Nat Rev Cancer. (2016) 16:582–98. doi: 10.1038/nrc.2016.73 27550820

[59] Mariathasan S Turley SJ Nickles D Castiglioni A Yuen K Wang Y . TGFbeta attenuates tumour response to PD-L1 blockade by contributing to exclusion of T cells. Nature. (2018) 554:544–8. doi: 10.1038/nature25501 29443960 PMC6028240

[60] Tauriello DVF Palomo-Ponce S Stork D Berenguer-Llergo A Badia-Ramentol J Iglesias M . TGFbeta drives immune evasion in genetically reconstituted colon cancer metastasis. Nature. (2018) 554:538–43. doi: 10.1038/nature25492 29443964

[61] Gabrilovich DI Nagaraj S . Myeloid-derived suppressor cells as regulators of the immune system. Nat Rev Immunol. (2009) 9:162–74. doi: 10.1038/nri2506 19197294 PMC2828349

[62] Cassetta L Pollard JW . Targeting macrophages: therapeutic approaches in cancer. Nat Rev Drug Discov. (2018) 17:887–904. doi: 10.1038/nrd.2018.169 30361552

[63] Veglia F Sanseviero E Gabrilovich DI . Myeloid-derived suppressor cells in the era of increasing myeloid cell diversity. Nat Rev Immunol. (2021) 21:485–98. doi: 10.1038/s41577-020-00490-y 33526920 PMC7849958

[64] Li K Shi H Zhang B Ou X Ma Q Chen Y . Myeloid-derived suppressor cells as immunosuppressive regulators and therapeutic targets in cancer. Signal Transduct Target Ther. (2021) 6:362. doi: 10.1038/s41392-021-00670-9 34620838 PMC8497485

[65] Woo SR Fuertes MB Corrales L Spranger S Furdyna MJ Leung MY . STING-dependent cytosolic DNA sensing mediates innate immune recognition of immunogenic tumors. Immunity. (2014) 41:830–42. doi: 10.1016/j.immuni.2014.12.015 25517615 PMC4384884

[66] Deng L Liang H Xu M Yang X Burnette B Arina A . STING-dependent cytosolic DNA sensing promotes radiation-induced type I interferon-dependent antitumor immunity in immunogenic tumors. Immunity. (2014) 41:843–52. doi: 10.1016/j.immuni.2014.10.019 25517616 PMC5155593

[67] Peng L Sferruzza G Yang L Zhou L Chen S . CAR-T and CAR-NK as cellular cancer immunotherapy for solid tumors. Cell Mol Immunol. (2024) 21:1089–108. doi: 10.1038/s41423-024-01207-0 39134804 PMC11442786

[68] Vivier E Rebuffet L Narni-Mancinelli E Cornen S Igarashi RY Fantin VR . Natural killer cell therapies. Nature. (2024) 626:727–36. doi: 10.1038/s41586-023-06945-1 38383621

[69] Kirk NA Kim KB Park KS . Effect of chromatin modifiers on the plasticity and immunogenicity of small-cell lung cancer. Exp Mol Med. (2022) 54:2118–27. doi: 10.1038/s12276-022-00905-x 36509828 PMC9794818

[70] Burr ML Sparbier CE Chan KL Chan YC Kersbergen A Lam EYN . An evolutionarily conserved function of Polycomb silences the MHC class I antigen presentation pathway and enables immune evasion in cancer. Cancer Cell. (2019) 36:385–401:e8. doi: 10.1016/j.ccell.2019.08.008 31564637 PMC6876280

[71] Bradley CA . PRC2-mediated MHC-I silencing drives immune evasion. Nat Rev Cancer. (2019) 19:664. doi: 10.1038/s41568-019-0219-4 31616077

[72] Gardner EE Lok BH Schneeberger VE Desmeules P Miles LA Arnold PK . Chemosensitive relapse in small cell lung cancer proceeds through an EZH2-SLFN11 axis. Cancer Cell. (2017) 31:286–99. doi: 10.1016/j.ccell.2017.01.006 28196596 PMC5313262

[73] Augert A Eastwood E Ibrahim AH Wu N Grunblatt E Basom R . Targeting NOTCH activation in small cell lung cancer through LSD1 inhibition. Sci Signal. (2019) 12(567):eaau2922. doi: 10.1126/scisignal.aau2922 30723171 PMC6530478

[74] Borromeo MD Savage TK Kollipara RK He M Augustyn A Osborne JK . ASCL1 and NEUROD1 reveal heterogeneity in pulmonary neuroendocrine tumors and regulate distinct genetic programs. Cell Rep. (2016) 16:1259–72. doi: 10.1016/j.celrep.2016.06.081 27452466 PMC4972690

[75] Pozo K Kollipara RK Kelenis DP Rodarte KE Ullrich MS Zhang X . ASCL1, NKX2-1, and PROX1 co-regulate subtype-specific genes in small-cell lung cancer. iScience. (2021) 24:102953. doi: 10.1016/j.isci.2021.102953 34466783 PMC8384902

[76] Hiatt JB Sandborg H Garrison SM Arnold HU Liao SY Norton JP . Inhibition of LSD1 with Bomedemstat sensitizes small cell lung cancer to immune checkpoint blockade and T-cell killing. Clin Cancer Res Off J Am Assoc For Cancer Res. (2022) 28:4551–64. doi: 10.1158/1078-0432.ccr-22-1128 35920742 PMC9844673

[77] Shue YT Drainas AP Li NY Pearsall SM Morgan D Sinnott-Armstrong N . A conserved YAP/Notch/REST network controls the neuroendocrine cell fate in the lungs. Nat Commun. (2022) 13:2690. doi: 10.1038/s41467-022-30416-2 35577801 PMC9110333

[78] Vanpouille-Box C Alard A Aryankalayil MJ Sarfraz Y Diamond JM Schneider RJ . DNA exonuclease Trex1 regulates radiotherapy-induced tumour immunogenicity. Nat Commun. (2017) 8:15618. doi: 10.1038/ncomms15618 28598415 PMC5472757

[79] Liang H Deng L Hou Y Meng X Huang X Rao E . Host STING-dependent MDSC mobilization drives extrinsic radiation resistance. Nat Commun. (2017) 8:1736. doi: 10.1038/s41467-017-01566-5 29170400 PMC5701019

[80] Saunders LR Bankovich AJ Anderson WC Aujay MA Bheddah S Black K . A DLL3-targeted antibody-drug conjugate eradicates high-grade pulmonary neuroendocrine tumor-initiating cells *in vivo*. Sci Transl Med. (2015) 7:302ra136. doi: 10.1126/scitranslmed.aac9459 26311731 PMC4934375

[81] Owen DH Giffin MJ Bailis JM Smit MD Carbone DP He K . DLL3: an emerging target in small cell lung cancer. J Hematol Oncol. (2019) 12:61. doi: 10.1186/s13045-019-0745-2 31215500 PMC6582566

[82] Rudin CM Pietanza MC Bauer TM Ready N Morgensztern D Glisson BS . Rovalpituzumab tesirine, a DLL3-targeted antibody-drug conjugate, in recurrent small-cell lung cancer: a first-in-human, first-in-class, open-label, phase 1 study. Lancet Oncol. (2017) 18:42–51. doi: 10.1016/s1470-2045(16)30565-4 27932068 PMC5481162

[83] Blackhall F Jao K Greillier L Cho BC Penkov K Reguart N . Efficacy and safety of rovalpituzumab tesirine compared with topotecan as second-line therapy in DLL3-high SCLC: results from the phase 3 TAHOE study. J Thorac Oncol. (2021) 16:1547–58. doi: 10.1016/j.jtho.2021.02.009 33607312

[84] Ahn MJ Cho BC Felip E Korantzis I Ohashi K Majem M . Tarlatamab for patients with previously treated small-cell lung cancer. N Engl J Med. (2023) 389:2063–75. doi: 10.1056/nejmoa2307980 37861218

[85] Mountzios G Sun L Cho BC Demirci U Baka S Gumus M . Tarlatamab in small-cell lung cancer after platinum-based chemotherapy. N Engl J Med. (2025) 393:349–61. doi: 10.1056/nejmoa2502099 40454646

[86] Monaca F Gomez-Randulfe I Torres-Jimenez J Agarwal M Liu SV Paz-Ares L . Antibody-drug conjugates in SCLC: DLL3, B7-H3, TROP2, and beyond. J Thorac Oncol. (2026), 103726. doi: 10.1016/j.jtho.2026.103726 41985715

[87] Wespiser M Gille R Perol M . Clinical progress of B7-H3 targeted antibody drug conjugate ifinatamab deruxtecan for small-cell lung cancer. Expert Opin Investig Drugs. (2025) 34:463–71. doi: 10.1080/13543784.2025.2512566 40418751

[88] Wiedemeyer WR Gavrilyuk J Schammel A Zhao X Sarvaiya H Pysz M . ABBV-011, a novel, calicheamicin-based antibody-drug conjugate, targets SEZ6 to eradicate small cell lung cancer tumors. Mol Cancer Ther. (2022) 21:986–98. doi: 10.1158/1535-7163.mct-21-0851 35642431 PMC9381089

